# Marijuana use among adolescent Asian Americans and Pacific islanders: Analysis of ethnic subgroup and gender differences

**DOI:** 10.1016/j.pmedr.2025.103016

**Published:** 2025-02-25

**Authors:** Ting Luo, Christopher M. Anderson, Jijiang Wang, Yue-Lin Zhuang, Shu-Hong Zhu

**Affiliations:** a The Herbert Wertheim School of Public Health and Human Longevity Science, University of California San Diego, 9500 Gilman Drive #0905, La Jolla, California, USA; b Moores Cancer Center, University of California San Diego, 9500 Gilman Drive #0905, La Jolla, California, USA

**Keywords:** Marijuana, Substance use, Asian American, Adolescence, Disaggregation, Gender, Pacific Islander

## Abstract

**Objective:**

To assess heterogeneity in marijuana use among adolescent Asian American and Pacific Islander (AAPI) subgroups.

**Methods:**

AAPI high school students (*n* = 31,071) participating in the 2019–2020 California Student Tobacco Survey were categorized by race/ethnicity and examined for ever and current (past-30-day) marijuana use. Descriptive statistics were used to describe marijuana use and harm perceptions by subgroups. Multiple logistic regression was used to compare subgroups on marijuana use with other demographics as covariates.

**Results:**

Current marijuana use rates by ethnic subgroups were: Chinese 4.2 %, Koreans 4.9 %, Indians 5.2 %, Vietnamese 6.0 %, Hmong 6.4 %, Pakistanis 6.6 %, Japanese 9.4 %, Filipinos 9.6 %, Cambodians 17.9 %, other monoethnic Asians 10.5 %, and Pacific Islanders 22.3 %. Current use rates for monoethnic, multiethnic, and multiracial AAPIs were 7.3 %, 9.1 %, and 18.5 %, respectively, with multiracial AAPIs using at a higher rate than monoethnic and multiethnic AAPIs (both *p*'s < 0.001). Among AAPIs overall, 11.3 % currently used marijuana. Females currently used at a higher rate than males, 10.8 % vs. 9.6 % (*p* = .011). Ever and current marijuana use were negatively correlated with perceptions that everyday and occasional use is harmful (all *p*'s < 0.001). Compared to Chinese students, all subgroups except Koreans and Pakistanis were more likely to use marijuana (all *p*'s < 0.05), with Filipinos, Japanese, Cambodians, and Pacific Islanders more than twice as likely (all *p*'s < 0.001).

**Conclusions:**

There was substantial heterogeneity in marijuana use rates among ethnic subgroups of AAPI adolescents. Several AAPI subgroups used marijuana at elevated rates. Gender-based trendlines among AAPI adolescents have crossed.

## Highlights

• Asian Americans and Pacific Islanders are often aggregated due to small samples.
• This study presents adolescent marijuana use rates disaggregated by subgroup.
• There was substantial heterogeneity in marijuana use rates among ethnic subgroups.
• Among Asian Americans and Pacific Islanders, girls use marijuana more than boys.

## 1 Introduction

Marijuana use during adolescence can interfere with brain development and lead to addiction (*National Academies of Sciences, Engineering, and Medicine*, 2017; Winters and Lee, 2008). Yet laws and norms concerning marijuana are becoming more relaxed in many developed nations and marijuana use is becoming more prevalent (United Nations Office on Drugs and Crime, 2022). A Monitoring the Future report found that in 2022, 5.0 % of eighth graders, 12.1 % of tenth graders, and 20.2 % of twelfth graders in the U.S. currently used marijuana, defined as use in the past 30 days (Miech et al., 2023). Similarly, a California Student Tobacco Survey study reported that 12.1 % of tenth graders and 18.0 % of twelfth graders in 2019–2020 currently used marijuana (same definition; Zhu et al., 2021). The study found considerable variability by race/ethnicity, however; current use rates were 6.6 % for Asian Americans, 14.9 % for Hispanics/Latinos, 17.1 % for Whites, 19.6 % for Blacks, 21.5 % for Pacific Islanders, and 22.4 % for American Indians/Alaska Natives (Zhu et al., 2021). The large difference in rates between Asian Americans and Pacific Islanders was notable, given that they are often treated as a single group (AAPI) when samples are too small to report reliable estimates for both (Wu and Blazer, 2015; Choi et al., 2024). Other studies have noted that while marijuana use rates are low for AAPIs compared to other races, they are much higher for adolescent Pacific Islanders than for their Asian American peers (Lee et al., 2021; Lowry et al., 2011).

Some studies have gone further in disaggregating marijuana use rates for AAPI adolescents (Austin, 1999; Otsuki, 2003; Wong et al., 2004). A California school survey conducted in 1989 and 1991 (AAPI *n* = 1003) reported that past-six-month marijuana use rates among ninth and eleventh graders were 2.9 % for Chinese, 5.0 % for Southeast Asians, 8.9 % for Japanese, 13.3 % for Koreans, 13.9 % for Filipinos, and 23.1 % for Pacific Islanders (Austin, 1999). Another California school survey conducted in 1996 (AAPI *n* = 4355) reported that current use rates for male and female twelfth graders, respectively, were 4.4 % and 1.9 % for Chinese, 6.8 % and 4.1 % for Japanese, 9.5 % and 2.7 % for Vietnamese, 9.7 % and 8.0 % for Koreans, and 18.6 % and 17.1 % for Filipinos (Otsuki, 2003). A third study based on surveys conducted in 1998–1999 (AAPI *n* ≥ 41,993) found that current use rates among ninth graders in California were 3.6 % for Chinese, 8.6 % for Japanese, 9.5 % for Filipinos, and 20.4 % for Pacific Islanders; and among tenth graders in Hawaii were 7.5 % for Chinese, 13.9 % for Japanese, 19.0 % for Filipinos, and 29.7 % for Native Hawaiians (Wong et al., 2004). These studies all defined current use as past-30-day use.

Since these studies were conducted, much has changed. The Asian American and Pacific Islander populations in the U.S. grew by 81 % and 61 %, respectively, from 2000 to 2019 (Budiman and Ruiz, 2021). By 2019–2020, AAPIs accounted for 12 % of California students (Anderson, 2021). California and many other states legalized recreational marijuana use (Hansen et al., 2023). Vaping became more popular than smoking (U.S. Department of Health and Human Services, 2016) and vaping devices made it easier for adolescents to use marijuana (Keyes et al., 2022). Given these changes, updated information on marijuana use among AAPI adolescents is needed.

This study aimed to produce reliable and updated prevalence estimates of marijuana use among AAPI adolescents disaggregated by ethnic subgroups, using data from the 2019–2020 California Student Tobacco Survey (CSTS), which had a large sample representative of California's public school population (Zhu et al., 2021b). The 2019–2020 CSTS was selected because sample sizes were sufficient to report on nine monoethnic AAPI subgroups, whereas subsequent surveys had much smaller samples. To our knowledge, this is the largest number of subgroups ever reported on regarding marijuana use among AAPI adolescents.

## 2 Methods

### 2.1 Sample and design

The CSTS was conducted from September 2019 to March 2020 in the eighth, tenth, and twelfth grades (*N* = 162,675). Details concerning CSTS implementation were published elsewhere (Zhu et al., 2021b). In brief, the survey applied a two-stage cluster sampling design to obtain a representative sample of California public school students. Primary sampling units were schools; secondary sampling units were classrooms. High schools and middle schools were sampled separately; the former were stratified into 35 regions before selection while the latter were sampled directly on a statewide basis. Of 3056 eligible schools, 608 were randomly selected to participate and 482 agreed to do so. The survey was completed in 369 schools, including 47 middle schools and 322 high schools. The response rate among eligible students was 68.3 % (Zhu et al., 2021b). Due to the limited number of eighth grade participants, it was not feasible to examine AAPI subgroups of middle school students. The study therefore focused on 31,071 AAPI high school students who provided information about their marijuana use.

The University of California San Diego Human Research Protections Program determined that the study conformed to its guidelines for the protection of human subjects concerning safety and privacy, and that the study was exempt from the requirement to request participant consent because it was based on a publicly available, anonymized database (#809580).

### 2.2 Measures

#### 2.2.1 Sociodemographic characteristics

Asian and Pacific Islander respondents were categorized as monoethnic AAPIs (i.e., solely Asian or Pacific Islander with a single ethnic background), multiethnic AAPIs (Asian or Pacific Islander with more than one ethnic background), or multiracial AAPIs (AAPI and another race, or AAPI and Hispanic). Monoethnic AAPIs were further categorized as Chinese, Filipinos, Vietnamese, Indians, Koreans, Hmong, Japanese, Pakistanis, Cambodians, other monoethnic Asians, or monoethnic Pacific Islanders. The latter two subgroups were comprised of ethnicities with samples too small to be analyzed separately (*n* < 200).

The survey assessed gender using the question, “How do you describe yourself?” with the options male, female, transgender male, transgender female, genderqueer, other, and “choose not to disclose.” For analysis, students were categorized as male, female, or other gender, with the latter comprised of all who selected an option other than male or female.

As a proxy measure for socioeconomic status, the survey asked, “Do either of your parents have a college degree?” with the options yes, no, and “I don't know.”

#### 2.2.2 Marijuana use

The survey asked if students had ever smoked, eaten, drunk, dabbed, or vaped marijuana, or used it in any other way. If so, they were classified as having ever used marijuana. Those who had ever used marijuana were asked if they had done so in the past 30 days. If so, they were classified as currently using marijuana.

#### 2.2.3 Marijuana harm perception

The survey asked students how harmful they thought everyday and occasional marijuana use were. Those who rated either frequency as harmful or extremely harmful were categorized as perceiving marijuana use at that frequency as harmful.

### 2.3 Statistical analysis

Descriptive statistics were used to describe demographic characteristics and marijuana use by subgroups. Point estimates were presented with 95 % confidence intervals, and chi-square tests were used to examine differences in marijuana use between and within groups. The association between students' perception of the harmfulness of marijuana use and the prevalence of marijuana use across ethnicities was calculated with the Pearson Correlation. Multiple logistic regression was used to compare subgroups of marijuana use with other demographics as covariates. All analyses took into account the complex multistage survey design, including stratification, clustering, and unequal weighting, with a finite population correction for sampling errors. Data were analyzed in 2023–2025 using SAS software 9.4.

## 3 Results

### 3.1 Sociodemographic characteristics

Table 1 displays weighted percentages for demographic characteristics by subgroups. Ethnicities are listed in declining order of size from Chinese (*n* = 4158) to Cambodians (*n* = 205). Ethnicities with fewer than 200 monoethnic students are aggregated as other monoethnic Asians (*n* = 805) or monoethnic Pacific Islanders (PIs) (*n* = 540). Monoethnic AAPIs (*n* = 17,282) represent all students reporting a single AAPI ethnicity. Multiethnic AAPIs (*n* = 3308) are those reporting more than one AAPI ethnicity. Multiracial AAPIs (*n* = 10,481) are those reporting both AAPI and non-AAPI race/ethnicities.

**Table 1:** Demographic Characteristics of Asian American and Pacific Islander High School Students by Subgroups: California, 2019–2020 (*n* = 31,071).

		Grade		Gender			Parental college degree^a^		
AAPI group	N (weighted %)	10	12	Male	Female	Other^b^	Yes	No	Unknown^c^
Chinese	4158 (12.6)	48.0	52.0	45.0	50.6	4.4	68.5	23.8	7.7
Filipino	3887 (13.3)	45.9	54.1	48.2	46.5	5.3	71.7	15.6	12.7
Vietnamese	2791 (9.3)	49.5	50.5	48.0	48.9	3.1	51.3	35.3	13.4
Indian	2401 (6.3)	51.3	48.7	46.7	51.6	1.7	84.7	10.5	4.8
Korean	1083 (4.1)	53.0	47.0	47.3	50.6	2.0	86.3	7.7	6.0
Hmong	808 (2.4)	47.5	52.5	46.9	48.3	4.8	36.5	45.3	18.3
Japanese	321 (1.2)	51.6	48.4	48.6	46.6	4.8	86.6	9.0	4.4
Pakistani	283 (0.8)	42.5	57.5	44.6	53.2	2.3	68.6	21.5	9.9
Cambodian	205 (0.6)	44.1	55.9	44.8	39.9	15.3	27.2	43.8	29.0
Other monoethnic Asian^d^	805 (2.6)	51.3	48.7	44.4	47.4	8.2	54.9	30.0	15.1
Other monoethnic PI^e^	540 (1.9)	47.1	52.9	35.6	38.4	26.1	29.0	37.8	33.1
Monoethnic AAPI^f^	17,282 (−)	48.5	51.5	46.5	48.6	5.0	66.1	22.6	11.3
Multiethnic AAPI^g^	3308 (10.5)	53.7	46.3	44.5	49.3	6.2	61.6	26.5	11.9
Multiracial AAPI^h^	10,481 (34.3)	55.1	44.9	43.6	44.6	11.8	57.8	28.6	13.6
Any AAPI^i^	31,071 (−)	51.4	48.6	45.3	47.3	7.4	62.8	25.1	12.2

*Notes:* AAPI = Asian American and Pacific Islander. PI = Pacific Islander. ^a^ Parental college degree: at least one parent has a college degree. ^b^ Other gender: respondent selected transgender male, transgender female, genderqueer, other, or “choose not to disclose,” or left the gender question blank. ^c^ Unknown parental college degree: respondent selected “I don't know” or left the question blank. ^d^ Other monoethnic Asian: a single Asian ethnicity other than Chinese, Filipino, Vietnamese, Indian, Korean, Hmong, Japanese, Pakistani, and Cambodian. ^e^ Other monoethnic PI: a single Pacific Islander ethnicity other than Filipino.

f Monoethnic AAPI: any one Asian American or Pacific Islander ethnicity. ^g^ Multiethnic AAPI: more than one Asian American or Pacific Islander ethnicity. ^h^ Multiracial AAPI: at least one Asian American or Pacific Islander ethnicity and at least one non-AAPI race or ethnicity. ^i^ Any AAPI: at least one Asian American or Pacific Islander ethnicity.

Tenth graders slightly outnumbered twelfth graders. Males (45.3 %) and females (47.3 %) were almost evenly divided. Another 7.4 % of AAPI students reported another gender. Among monoethnic, multiethnic, and multiracial AAPIs, 5.0 %, 6.2 %, and 11.8 %, respectively, reported another gender. Among ethnic subgroups, the proportions reporting another gender ranged from 1.7 % of Indians to 26.1 % of Pacific Islanders. Nearly two thirds of respondents, 62.8 %, had a parent with a college degree, but proportions ranged widely, from 27.2 % of Cambodians to 86.6 % of Japanese.

### 3.2 Marijuana use rates

Table 2 shows marijuana use rates by subgroups. Overall, nearly a quarter of AAPI students (23.2 %) had ever used marijuana and 11.3 % currently used it. Ever use rates ranged from 9.7 % of Chinese to 41.7 % of Pacific Islanders. For monoethnic, multiethnic, and multiracial AAPIs, ever use rates were 16.1 %, 20.6 %, and 35.4 %, respectively (all *p*'s < 0.001). Current use rates were 4.2 % for Chinese, 4.9 % for Koreans, 5.2 % for Indians, 6.0 % for Vietnamese, 6.4 % for Hmong, 6.6 % for Pakistanis, 9.4 % for Japanese, 9.6 % for Filipinos, 17.9 % for Cambodians, 10.5 % for other monoethnic Asians, and 22.3 % for other monoethnic Pacific Islanders. For monoethnic, multiethnic, and multiracial AAPIs, current use rates were 7.3 %, 9.1 %, and 18.5 %, respectively (all *p*'s < 0.001); multiracial AAPIs were significantly more likely to report current marijuana use than either monoethnic or multiethnic AAPIs (both *p*'s < 0.001). Non-overlapping 95 % confidence intervals indicate statistically significant differences between subgroups. For example, compared to Chinese students, most other ethnic subgroups had significantly higher ever and current use rates.

**Table 2:** Marijuana Use Among Asian American and Pacific Islander High School Students by Subgroups: California, 2019–2020 (n = 31,071).

		Ever use^a^		Current use^b^	
AAPI group	n	%	95 % CI	%	95 % CI
Chinese	4158	9.7	(8.6–10.8)	4.2	(3.5–4.9)
Korean	1083	12.2	(9.1–15.2)	4.9	(3.7–6.2)
Indian	2401	10.6	(9.3–11.9)	5.2	(4.3–6.2)
Vietnamese	2791	14.3	(12.7–15.9)	6.0	(4.9–7.1)
Hmong	808	15.5	(12.9–18.0)	6.4	(4.8–8.0)
Pakistani	283	12.4	(8.0–17.0)	6.6	(3.5–9.7)
Japanese	321	15.4	(11.8–18.9)	9.4	(5.7–13.2)
Filipino	3887	22.5	(19.8–25.2)	9.6	(8.3–10.9)
Cambodian	205	31.3	(24.4–38.2)	17.9	(11.5–24.3)
Other monoethnic Asian^c^	805	18.8	(15.8–21.8)	10.5	(7.8–13.1)
Other monoethnic PI^d^	540	41.7	(35.9–47.5)	22.3	(17.2–27.4)
Monoethnic AAPI^e^	17,282	16.1	(14.8–17.4)	7.3	(6.7–8.0)
Multiethnic AAPI^f^	3308	20.6	(18.7–22.6)	9.1	(7.9–10.3)
Multiracial AAPI^g^	10,481	35.4	(33.9–36.9)	18.5	(17.3–19.7)
Any AAPI^h^	31,071	23.2	(21.8–24.6)	11.3	(10.5–12.2)

*Notes:* AAPI = Asian American and Pacific Islander. CI = confidence interval. PI = Pacific Islander. ^a^ Ever use: any use during respondent's lifetime. ^b^ Current use: any use during the past 30 days. ^c^ Other monoethnic Asian: Asian ethnicity other than Chinese, Korean, Indian, Vietnamese, Hmong, Pakistani, Japanese, Filipino, and Cambodian. ^d^ Other monoethnic PI: Pacific Islander ethnicity other than Filipino. ^e^ Monoethnic AAPI: any one Asian American or Pacific Islander ethnicity. ^f^ Multiethnic AAPI: more than one Asian American or Pacific Islander ethnicity. ^g^ Multiracial AAPI: at least one Asian American or Pacific Islander ethnicity and at least one non-AAPI race or ethnicity. ^h^ Any AAPI: at least one Asian American or Pacific Islander ethnicity.

A multivariable analysis of marijuana use rates among AAPI subgroups, controlling for grade, gender, and parental education, confirmed that race/ethnicity independently predicted marijuana use. With Chinese students serving as the reference group due to their large size and low use rates, all other subgroups were significantly more likely to use marijuana currently except Koreans (odds ratio [OR] = 1.31; *p* = .100) and Pakistanis (OR = 1.60; *p* = .108). Subgroups more than twice as likely include Japanese (OR = 2.54), Filipinos (OR = 2.41), Cambodians (OR = 4.10), other monoethnic Asians (OR = 2.58), and monoethnic Pacific Islanders (OR = 5.05; all *p*'s < 0.001). Multiethnic AAPIs (OR = 2.30) and multiracial AAPIs (OR = 5.05) were also more likely than Chinese students to use marijuana currently (both *p*'s < 0.001). Similar patterns were observed regarding ever use.

### 3.3 Marijuana use by gender

**Fig 1:**
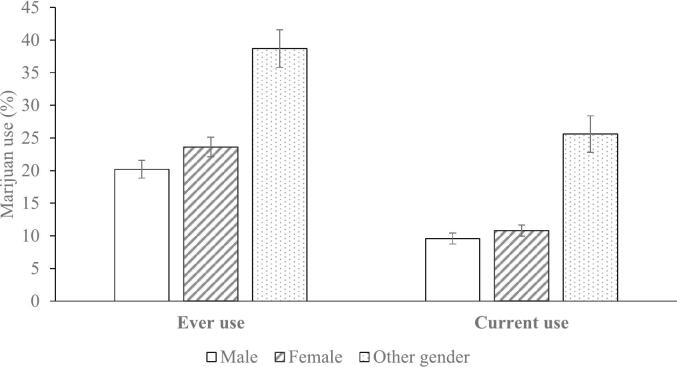
Gender Differences in Marijuana Use Among Asian American and Pacific Islander High School Students: California, 2019–2020 (n = 31,071).

There was substantial variation in marijuana use by gender. As shown in Fig. 1, females were significantly more likely than males to have ever used marijuana, 23.6 % vs. 20.2 % (*p* < .001). Students of other gender had an even higher ever use rate, 38.7 %, than either males or females (both *p*'s < 0.001). Current use rates showed the same pattern: females were more likely than males to use currently, 10.8 % vs. 9.6 % (*p = .*011), and students of other gender used currently at a higher rate, 25.6 %, than either males or females (both *p*'s < 0.001).

A multivariable analysis, controlling for the effects of ethnicity, grade, and parental education, confirmed that females were 16 % more likely than males to use marijuana currently (OR = 1.16) and students of other gender were 156 % more likely than males to use it currently (OR = 2.56; both *p*'s < 0.001).

### 3.4 Marijuana harm perceptions

Table 3 shows the proportions of respondents perceiving everyday and occasional marijuana use as harmful. Overall, 69.7 % of AAPI students perceived everyday use as harmful, and 54.3 % perceived occasional use as harmful. Among ethnic subgroups, the proportions perceiving harm from everyday use ranged from 57.8 % of Pacific Islanders to 82.8 % of Japanese. The corresponding proportions for monoethnic, multiethnic, and multiracial AAPIs were 76.6 %, 71.7 %, and 57.9 %, respectively. The proportions perceiving harm from occasional use ranged from 48.7 % of Pacific Islanders to 70.0 % of Japanese, and the corresponding proportions for monoethnic, multiethnic, and multiracial AAPIs were 61.3 %, 56.5 %, and 42.1 %, respectively. As in Table 2, non-overlapping 95 % confidence intervals indicate statistically significant differences.

**Table 3:** Perceived Harm of Using Marijuana Among Asian American and Pacific Islander High School Students by Subgroups: California, 2019–2020 (n = 31,071).

		Perceived harm of using marijuana^a^			
		Everyday use		Occasional use	
AAPI group	n	%	95 % CI	%	95 % CI
Chinese	4158	81.6	(80.5–82.8)	66.5	(64.8–68.1)
Korean	1083	79.5	(77.1–82.0)	65.7	(62.5–68.8)
Indian	2401	80.7	(78.6–82.8)	65.2	(62.7–67.7)
Vietnamese	2791	77.1	(75.4–78.8)	59.9	(58.1–61.7)
Hmong	808	79.8	(76.6–83.0)	63.5	(58.2–68.8)
Pakistani	283	79.4	(74.2–84.7)	62.2	(55.4–69.1)
Japanese	321	82.8	(79.2–86.3)	70.0	(63.9–76.0)
Filipino	3887	72.2	(70.1–74.3)	56.0	(53.6–58.3)
Cambodian	205	58.2	(51.8–64.5)	49.3	(42.6–56.0)
Other monoethnic Asian^b^	805	69.8	(65.6–74.1)	59.0	(55.0–62.9)
Other monoethnic PI^c^	540	57.8	(52.4–63.2)	48.7	(43.2–54.3)
Monoethnic AAPI^d^	17,278	76.6	(75.5–77.6)	61.3	(60.2–62.5)
Multiethnic AAPI^e^	3308	71.7	(70.0–73.4)	56.5	(54.4–58.6)
Multiracial AAPI^f^	10,481	57.9	(56.5–59.2)	42.1	(40.8–43.5)
Any AAPI^g^	31,071	69.7	(68.5–70.8)	54.3	(53.0–55.5)

*Notes:* AAPI = Asian American and Pacific Islander. CI = confidence interval. PI = Pacific Islander. ^a^ Perceived harm of using marijuana: respondent rated marijuana use (everyday or occasional) as harmful or extremely harmful. ^b^ Other monoethnic Asian: Asian ethnicity other than Chinese, Korean, Indian, Vietnamese, Hmong, Pakistani, Japanese, Filipino, and Cambodian. ^c^ Other monoethnic PI: Pacific Islander ethnicity other than Filipino. ^d^ Monoethnic AAPI: any one Asian American or Pacific Islander ethnicity. ^e^ Multiethnic AAPI: more than one Asian American or Pacific Islander ethnicity. ^f^ Multiracial AAPI: at least one Asian American or Pacific Islander ethnicity and at least one non-AAPI race or ethnicity. ^g^ Any AAPI: at least one Asian American or Pacific Islander ethnicity.

Perception of harm was negatively correlated with the prevalence of marijuana use across ethnicities (Table 2, Table 3). In general, groups that perceived everyday or occasional marijuana use as more harmful had lower ever and current use rates (all *p*'s < 0.001).

## 4 Discussion

In adolescent substance use research, Asian Americans and Pacific Islanders of diverse ethnicities are often aggregated. AAPIs collectively have low substance use rates relative to other racial groups, but aggregation can mask significant subgroup differences. This study, capitalizing on a school survey with a robust sample of AAPI adolescents, found substantial heterogeneity in marijuana use among monoethnic subgroups. Chinese, Koreans, Indians, Vietnamese, Hmong, and Pakistanis had below-average current use rates (4.2 %–6.6 %) while Japanese, Filipinos, Cambodians, and other monoethnic Asians and Pacific Islanders had above-average rates (9.4 %–22.3 %). Many subgroup differences were statistically significant. Moreover, multiracial AAPIs were significantly more likely to use marijuana than monoethnic and multiethnic AAPIs. This study thus provides a detailed view of marijuana use among AAPI adolescents and confirms that use rates vary widely by subgroups.

The ordering of marijuana use rates in this study is consistent with those of earlier studies (Austin, 1999; Otsuki, 2003; Wong et al., 2004); i.e., Chinese students had the lowest rates, Pacific Islanders had the highest rates, and Filipino rates were midway between these two. The consistency across studies suggests that there are meaningful, predictable differences among subgroups of AAPI adolescents regarding their propensity to use marijuana. The current study found that Korean, Indian, Vietnamese, Hmong, and Pakistani adolescents, groups seldom reported in the literature, had low marijuana use rates close to those of Chinese adolescents; that Japanese and Filipino adolescents again had rates significantly above average for AAPIs; that Cambodian adolescents, another group seldom reported, had rates close to those of Pacific Islanders; and that Pacific Islanders again ranked highest in marijuana use among AAPI subgroups. Subgroup differences may partly be due to varying community characteristics such as marijuana use rates and attitudes toward marijuana use in ancestral homelands and degrees of community acculturation. Thai et al. (2010) have shown that personal and interpersonal factors including individual acculturation, peer substance use, and academic achievement are also important in determining adolescents' likelihood of using marijuana.

Interestingly, marijuana use rates of multiethnic AAPIs in this study were comparatively high but within the range of several monoethnic subgroups, suggesting an averaging effect across ethnic subgroups. Multiethnic use rates were much closer to monoethnic use rates than to multiracial use rates, suggesting that having AAPI parents confers some protection against adolescent marijuana use, even if the parents have different AAPI ethnicities. Further research is needed to confirm this hypothesis. It should be noted that multiethnic AAPIs were a large group, accounting for more than a tenth (10.5 %) of AAPI adolescents in this study.

To our knowledge, this is the first study to report on marijuana use among multiracial AAPI adolescents. This was an even larger group than multiethnic AAPIs, accounting for more than a third (34.3 %) of AAPIs. Students in this group were among the most likely to use marijuana. Previous studies showed that multiracial adolescents in general are more likely to use marijuana than monoracial adolescents, including monoracial Asians (Thai et al., 2010; Choi et al., 2006; Choi et al., 2012); these studies reported use rates two to three times higher among multiracial teens than among monoracial AAPI teens. Researchers have noted that multiracial teens experience unique conditions and stressors that make them more likely to use marijuana (Goings et al., 2018), and that their more diverse peer networks expose them to higher rates of marijuana use (Choi et al., 2012). Goings et al. (2018) observed that multiracial teens are themselves a diverse group who can be further divided into subgroups varying significantly with respect to their levels of psychosocial protection and risk; for example, multiracial teens in low-income, one-parent households are less protected than those in higher-income, two-parent households. This study confirmed that students in lower-income households (as indicated by parental education, a proxy for socioeconomic status) were more likely to use marijuana. Multiracial AAPI teens may differ from other multiracial teens in important ways that could influence their likelihood of using marijuana, such as regarding cultural or parental attitudes toward substance use. In any case, multiracial AAPI teens in this study used marijuana at lower rates than multiracial teens in other studies (Choi et al., 2006; Choi et al., 2012), but at higher rates than most other AAPI subgroups in the study. In fact, multiracial AAPIs were more than twice as likely to use marijuana as monoracial AAPIs. If the hypothesis that adolescent marijuana use is influenced by the ethnicities of both parents is correct, the higher use rates observed among multiracial AAPIs may be due partly to higher prevalence rates among non-AAPI racial groups. More research is needed in this area.

The study also found that gender independently predicted marijuana use, with females 16 % more likely than males to use marijuana currently. Historically, male adolescents have used marijuana at higher rates than female adolescents, but this difference has been shrinking for some time (Johnson et al., 2015; Gu et al., 2021). Johnson et al. (2015), analyzing National Youth Risk Behavior Survey data for 1999–2009, observed that the adolescent gender gap in marijuana use was “quickly becoming nonexistent.” Gu et al. (2021) confirmed this change using Monitoring the Future data for 1991–2018, finding that the adolescent male and female trendlines had grown closer over several years, converged in 2016, then crossed over. Echoing these changes, the school survey on which the current study was based found that marijuana use rates in 2019–2020 were higher for females than for males (Zhu et al., 2021). These findings are remarkable given the greater stigma that females who use substances have historically borne relative to males (Meyers et al., 2021)—stigma that can be especially strong in Asian communities (Bradby, 2007). The study found even higher marijuana use rates among students not identifying as male or female. These gender-related developments occurred in a context in which the adult gender gap was also closing, likely due to the legalization of recreational marijuana and to broader societal changes in gender norms (Matheson and Le Foll, 2023). Research should continue monitoring gender-based changes in marijuana use among AAPIs, for both adolescents and adults.

The study found that harm perceptions regarding everyday and occasional marijuana use were negatively correlated with marijuana ever and current use rates. Concerningly, however, nearly a third of AAPI students (31.3 %) saw little harm in everyday marijuana use, and nearly half (45.7 %) saw little harm in occasional use. Perceptions also varied by subgroup, with Pacific Islanders, Cambodians, and multiracial AAPIs particularly unlikely to view marijuana use as harmful.

The study's findings have implications for clinicians, researchers, educators, and public health officials. First, subgroups of AAPI adolescents may have widely diverging marijuana use rates, with some much higher than the average for all AAPI adolescents, supporting the need for better screening by pediatricians and school health personnel to identify and intervene with AAPI youth who use marijuana, particularly in subgroups with higher use rates. Second, assumptions that girls are less likely to use marijuana than boys may be incorrect, given that a reversal of this trend has been observed not just among adolescents in general, but among AAPI adolescents in particular. Research is needed on the drivers of these gender-based changes. Finally, three in ten AAPI adolescents saw little harm in everyday marijuana use, suggesting that educators and public health officials have work to do to better inform young people of the risks of marijuana use.

### 4.1 Strengths and limitations

Strengths of this study included an AAPI sample (*n* = 31,071) sufficient to produce reliable estimates for multiple ethnic subgroups, a random sampling design, and multivariable analysis, increasing confidence in the findings. Moreover, the study provided marijuana use rates for multiethnic and multiracial AAPIs, two rarely examined subgroups.

The study also had limitations. Although the survey was representative of public high school students in California, its findings may not be generalizable to students elsewhere. Additional relevant factors that could help explain subgroup differences in marijuana use (e.g., immigrant status, acculturation, experiences of discrimination) were not measured. Although the sample was sufficient to conduct subgroup analyses, some subgroups were much smaller than others and therefore exhibited fewer significant differences from other subgroups, and many were still too small to disaggregate, including Fijians, Samoans, Tongans, and other Pacific Islander subgroups.

## 5 Conclusions

This study with a representative sample of AAPI adolescents in California contributes to the literature by providing disaggregated marijuana use rates for multiple ethnic subgroups. The results demonstrate that, while AAPI adolescents collectively use marijuana at low rates compared to their non-AAPI peers, there is substantial heterogeneity among AAPI subgroups. The study also shows that among AAPI adolescents, females now use marijuana at higher rates than males while students of other gender use at even higher rates.

## Declaration of generative AI in scientific writing

No generative artificial intelligence (AI) tools or AI-assisted technologies were used in the writing process of this manuscript, ensuring that all content, including language and presentation, was produced without such assistance, in full compliance with Elsevier's AI policy for authors.

## CRediT authorship contribution statement

**Ting Luo:** Writing – review & editing, Writing – original draft, Investigation, Formal analysis, Conceptualization. **Christopher M. Anderson:** Writing – review & editing, Investigation. **Jijiang Wang:** Writing – review & editing, Methodology, Investigation, Formal analysis, Data curation. **Yue-Lin Zhuang:** Writing – review & editing, Methodology, Investigation, Formal analysis. **Shu-Hong Zhu:** Writing – review & editing, Supervision, Investigation, Funding acquisition, Conceptualization.

## Declaration of competing interest

This work was supported by the 10.13039/100005002California Department of Public Health [grant number CDPH-22-11207]. The study sponsor had no involvement in the study design; collection, analysis, and interpretation of data; writing of the report; or the decision to submit the article for publication.

## Data Availability

The data underlying the results presented in the study are owned by a third party. Data from the California Student Tobacco Survey (CSTS) are owned by the California Department of Public Health. The data are available to interested researchers upon request. For CSTS data, please contact Dr. Xueying Zhang of the California Department of Public Health at Xueying.Zhang@cdph.ca.gov.
